# Development of *Chrysomya megacephala* at constant temperatures within its colony range in Yangtze River Delta region of China

**DOI:** 10.1080/20961790.2017.1403007

**Published:** 2017-12-21

**Authors:** Yingna Zhang, Yu Wang, Lijun Yang, Luyang Tao, Jiangfeng Wang

**Affiliations:** Department of Forensic Medicine, Soochow University, Suzhou, China

**Keywords:** Forensic science, forensic entomology, oriental latrine fly, development time, isomorphen/isomegalen diagram, thermal summation model, intra-puparial development

## Abstract

*Chrysomya megacephala* (Fabricius, 1794) is the most abundant and predominant species which arrives and colonizes a cadaver first in most parts of China. Therefore, its growth and development patterns have great implications in the estimation of the minimum postmortem interval (PMI_min_). In this study, *C. megacephala* was collected from the Yangtze River Delta region and reared at seven constant temperatures ranging from 16 °C to 34 °C. The developmental duration and accumulated degree hours, larval body length and morphological changes of *C. megacephala* were examined. Furthermore, we constructed three developmental models, isomorphen diagram, isomegalen diagram and thermal summation model, which can be used for estimating PMI_min_. The developmental durations of *C. megacephala* at 16 °C, 19 °C, 22 °C, 25 °C, 28 °C, 31 °C and 34 °C are (794.8 ± 14.7), (533.2 ± 10.1), (377.8 ± 16.8), (280.8 ± 15.1), (218.9 ± 8.5), (190.8 ± 10.1) and (171.8 ± 6.8) h, respectively. The developmental threshold temperature *D*_0_ is (11.41 ± 0.32) °C, and the thermal summation constant *K* is (3 418.7 ± 137.0) degree hours. Regression analysis was conducted to obtain equations of the variation in larval body length with time after hatching, and variation in time after hatching with body length. Moreover, our study divides the intra-puparial morphological changes of *C. megacephala* into 11 sub-stages, and provides the time range experienced by each sub-stage. The results of this study provide fundamental development data for the use of *C. megacephala* in PMI_min_ estimations.

## Introduction

Among the various necrophagous insects, blowflies (Diptera: Calliphoridae) are usually the first to colonize cadavers. The developmental stages of their offspring, larval body length/weight and intra-puparial morphological changes can provide accurate estimation of the minimum postmortem interval (PMI_min_) [1,2]. Therefore, it is particularly important to establish accurate basic developmental data for blowfly species.

The development of blowflies is relatively well characterized in forensic entomology studies [3–11]. For some of the globally distributed species, such as *Calliphora vicina* (Robineau-Desvoidy, 1830), *Lucilia sericata* (Meigen, 1826) and *Phormia regina* (Meigen, 1826), basic developmental data for different regions have been established using flies from different populations [12–15]. There are several developmental models that are used to estimate PMI_min_, such as isomorphen/isomegalen diagram and thermal summation model [14,16].

Isomorphen diagram is a scatterplot that models the duration of developmental events against temperature. Isomegalen diagram is a contour plot that contains three variables: the duration after hatching, different constant temperatures and larval size. Each contour represents the larval size (body length or width or weight) indicating the developmental duration under different temperatures [2]. In the thermal summation model, the *x*-axis is the developmental duration and the *y*-axis is the developmental accumulated degree days. The thermal summation model and equation (*y* = *ax* + *b*) of a developmental stage can be obtained by a linear regression analysis of the relationship between the duration and accumulated degree days/hours of each developmental stage. The slope in the equation is the developmental threshold temperature (*D*_0_) of the species, and the intercept *b* is the thermal summation constant *K* at a particular developmental stage [2,17].

Despite the large number of studies on development of blowflies, information is still lacking for populations from different geographical regions [12,18]. Furthermore, the differences in experimental feeding methods or feeding substrate may also lead to discrepancy in developmental time. Therefore, it is of great importance to establish basic development data for different regions to improve estimation of PMI_min_.

*Chrysomya megacephala* (Fabricius, 1794), also known as the oriental latrine fly, is mainly distributed in Australia and the Pacific [19]. Since the 1970s, the distribution of this species has gradually expanded and now is a globally distributed species [20]. In some areas, *C. megacephala* has become predominant over the local species [21]. Previous studies have shown that *C. megacephala* is one of the first flies to arrive at a dead body [22–26], and its overly large population size makes *C. megacephala* the absolute dominant species on the corpse [27,28].

Many studies have already investigated the development of *C. megacephala*, including development at constant temperatures [19,29–32], fluctuating temperatures [28,33] as well as the effects of different feeding tissues [34] or drugs [35,36]. The results show developmental plasticity in *C. megacephala*, and that different populations have differences in development. Since China runs through the Palaearctic and Oriental regions, its complex geography may lead to divergence of different local populations. *Chrysomyamegacephala* is distributed throughout China and is the most important outdoor necrophagous species [37,38]. Thus, acquisition of more precise developmental data, especially from different populations of this species, is of forensic significance. This article reports the developmental data from the Yangtze region that allows a more accurate PMI_min_ estimation in this region.

## Materials and methods

### Colony establishment

*Chrysomyamegacephala* was collected from pig carcasses placed in a field near the Forensic Autopsy Centre of Suzhou located in the centre of the Yangtze River Delta, China (31°21' N, 120°53' E) between 2015 and 2016. The post-feeding larvae were collected into an insect rearing box (NoA3, Huamei, 32 cm × 22 cm × 10 cm) filled with 2 cm of wet sand, along with a 3 cm × 5 cm ventilating nylon mesh window at the centre of the box. After pupation, the cap of the box was removed, and the rearing box was placed into a larger nylon insect rearing cage sized 200 cm × 100 cm × 100 cm because *C. megacephala* need more space to complete mating and oviposition based on our previous rearing experience. The rearing cage was kept at room temperature (about 25.0 °C), with 70% humidity and natural light. After eclosion, adults were fed with water and a 1:1 mixture of milk powder and sugar. Five days after eclosion, a culture dish (14 cm in diameter) containing 20 g of fresh pork was placed into the cage to induce oviposition. Eggs were collected and reared for three generations to establish a purebred colony for the subsequent study. Adult *C. megacephala* was identified using the identification key by Fan [39]. The number of adult *C. megacephala* in the cage was maintained at 3000–4000 during the study.

### Monitoring of developmental duration and measurement of larval body length

Fresh pork (20 g) was placed on a culture dish (10 cm diameter), which was placed into the insect-rearing cage to induce oviposition. Egg masses containing about 1500 eggs that were deposited on the pork within 1 h were carefully divided into six portions containing approximately 250 eggs per portion, each of which was placed into a 10 cm culture dish containing 20 g of fresh pork. These culture dishes were then moved into an insect rearing box (32 cm × 22 cm × 10 cm) covered with wet sand. The egg masses were kept separately to avoid increase in temperature caused by accumulation of larvae, which may impact experimental results. The insect rearing box was placed into the LHP-300H incubator (Yingmin Co. Ltd, Suzhou, China) at constant temperatures of 16 °C, 19 °C, 22 °C, 25 °C, 28 °C, 31 °C and 34 °C with 75% humidity and 12:12 light/dark cycle. Fresh lean pork was replenished 1–3 times a day based on the consumption by the larvae. Fresh lean pork was added uniformly throughout the culture dish to ensure that the larvae can be evenly distributed during food intake.

The eggs were observed every 1 h. After hatching, eight larvae were sampled every 4 h until pupation. The sampled larvae were treated in 90 °C hot water for 30 s, and stored in 75% ethanol. Larval samples were examined under a Zeiss 2000-C stereomicroscope to determine the larval instar based on the number of clefts in the posterior spiracle. During the pupal stage, observations were conducted every 4 or 8 h until eclosion. The time of hatching, pupation and eclosion were recorded during the experiment. The body length of the sampled larvae was measured using a digital vernier caliper with a precision of 0.01 mm (Shengong, Shanghai). Each experiment was repeated four times for each temperature in different incubators.

### Observation of intra-puparial development

Eggs were obtained using the methods described earlier and incubated at constant temperatures of 16 °C, 22 °C, 28 °C and 34 °C with 75% humidity 12:12 light/dark cycle. Upon pupation, 10 pupae from each temperature range were sampled every 8 h until adult eclosion, and treated as described previously [40,41]. The intra-puparial morphological changes, which are pupal characteristics that are used to classify the age of a pupa, were identified and imaged with a digital camera (Nikon D700) [42].

### Data analysis

Data analysis was performed using Origin Pro 8.6. The effect of temperature on duration of development was analysed using one-way ANOVA. The relationship between the larval body length and time after hatching was examined by nonlinear regression analysis using “larval body length” as the independent variable and “time after hatching” as the dependent variable, and vice versa, in order to model the equation for estimating the PMI_min_ [4,43]. The relationship between developmental duration and accumulated degree hours (ADH) in each developmental stage and total developmental process was analysed using the revised regression model proposed by Ikemoto and Takai [17], where the slope and intercept of the linear regression equation represent the developmental threshold temperature *D*_0_ and thermal summation constant *K* of *C. megacephala*, respectively.

## Results

### Developmental duration and construction of isomorphen diagram

Between 16 °C and 34 °C, the developmental duration of eggs, first instar, second instar and third instar larvae, and pupae decreases with higher temperatures, and the total developmental duration is shortened from 794.8 h at 16.0 °C to 171.8 h at 34.0 °C, indicating that the developmental duration of *C. megacephala* is significantly affected by temperature (Table 1). There are no significant differences in the developmental durations of larvae in the first instar stage, the second instar stage, third stage and the pupal stage between 28 °C and 31 °C, and between 31 °C and 34 °C. Nevertheless, the entire developmental process is significantly different between different temperatures.

**Table 1.** Mean (±SD) development duration (h) of *Chrysomya megacephala* at seven constant temperatures.

Developmental stages (°C)	Egg	First instar	Second instar	Third instar	Pupa	Total duration
16	38.9 ± 2.1^a^	81.3 ± 4.1^a^	84.0 ± 3.8^a^	248.2 ± 5.2^a^	342.5 ± 17.2^a^	794.8 ± 14.7^a^
19	27.2 ± 2.3^b^	57.3 ± 4.0^b^	58.7 ± 6.1^b^	148.2 ± 14.4^b^	241.8 ± 13.9^b^	533.2 ± 10.1^b^
22	19.2 ± 1.6^c^	35.2 ± 6.1^c^	40.2 ± 6.1^c^	108.6 ± 8.3^c^	174.7 ± 8.6^c^	377.8 ± 16.8^c^
25	14.8 ± 0.9^d^	28.6 ± 2.3^d^	32.2 ± 2.3^d^	68.0 ± 8.3^d^	138.8 ± 9.1^d^	280.8 ± 15.1^d^
28	12.2 ± 0.9^e^	18.0 ± 2.3^e^	22.5 ± 4.2^e^	61.2 ± 4.0^d^	105.2 ± 6.1^e^	218.9 ± 8.5^e^
31	10.3 ± 1.0^ef^	13.3 ± 2.3^ef^	16.2 ± 2.3^ef^	56.8 ± 3.3^de^	94.6 ± 4.2^ef^	190.8 ± 10.1^f^
34	8.5 ± 0.5^f^	11.3 ± 1.2^f^	13.3 ± 2.3^f^	49.6 ± 2.3^e^	89.3 ± 4.9^f^	171.8 ± 6.8^g^

Note: Values within the same column followed by the same superscript letter do not differ significantly from each other based on a one-way ANOVA + LSD test at *P* < 0.05.

**Figure 1.**
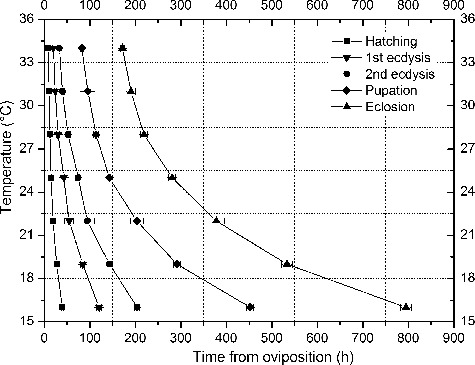
Isomorphen diagram of *Chrysomya megacephala*. The duration of each development event (hatching, first ecdysis, second ecdysis, wandering, pupation and eclosion) plotted with the time from oviposition to the onset of each event. Each curve corresponds to a developmental event, and the error bar is the standard deviation of each event.

The isomorphen diagram (Figure 1) was established based on the length of time (*x*-axis) for different developmental events at different constant temperatures (*y*-axis). In the temperature range of 16 °C–34 °C, the duration of each developmental event (hatching, first ecdysis, second ecdysis, pupation and eclosion) gradually shortens as the temperature increased, and the distance between each curve also shortened with increasing temperatures.

### Thermal summation model

**Figure 2.**
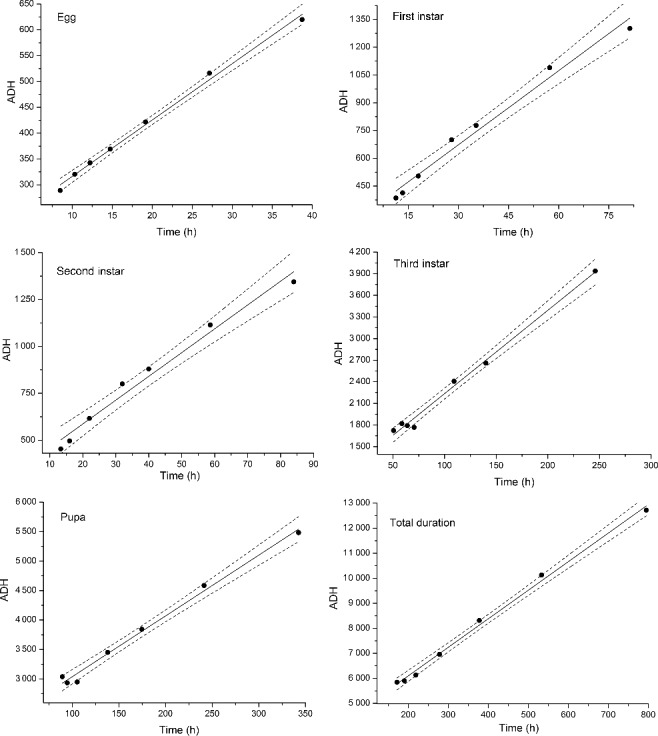
Thermal summation models of total developmental stages of *Chrysomya megacephala.* • indicates data used in the regression analysis.

A total of six thermal summation models were constructed from the linear regression analysis of the relationship between the development duration (*x*-axis) and ADH (*y*-axis) at each developmental stage and the entire developmental process (Figure 2). The coefficient of determination (*R*^2^) of the equation of each thermal summation model is ≥0.97, indicating relatively good fit of these linear models. The developmental threshold temperature and thermal summation constant of each development stage and the total development process were determined from each thermal summation model (Table 2). The developmental threshold temperature *D*_0_ and the thermal summation constant *K* of the entire developmental process is (11.41 ± 0.32) °C and (3 418.7 ± 137.0) degree hours, respectively.

**Table 2.** Mean (±SE) of developmental threshold temperatures (*D*_0_) and thermal summation constants (*K*) for five developmental stages and the total development period of *Chrysomya megacephala*, and the coefficient of determination (*R*^2^) of thermal summation models.

	*K* (degree hours)		*D*_0_ (°C)		
Developmental stages	Mean	SE	Mean	SE	*R*^2^
Egg	207.2	7.2	10.91	0.34	0.99
First instar	272.0	33.9	13.35	0.79	0.98
Second instar	332.8	37.3	12.68	0.83	0.97
Third instar	1077.6	56.2	11.57	0.45	0.99
Pupa	2007.2	81.4	10.31	0.43	0.99
Total duration	3418.7	137.0	11.41	0.32	0.99

### Larval body length changes and construction of isomegalen diagram

**Figure 3.**
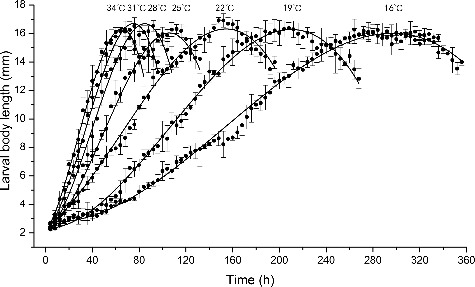
Larval body length changes of *Chrysomya megacephala* over time after hatching at different temperatures.

Changes in larval body length of *C. megacephala* at different temperatures are shown in Figure 3, where the larval developmental rate increases rapidly with increasing temperature increased. Between 16 °C and 25 °C, the development rate is significantly different between each temperature, but the difference is less pronounced as the temperature increased to 28 °C–34 °C. The mean maximum larval body length at 16 °C, 19 °C, 22 °C, 25 °C, 28 °C, 31°C and 34 °C are 16.2, 16.3, 17.0, 16.3, 16.5, 16.4 and 16.2 mm, respectively.

The equations in Table 3 describe the changes in larval body length (*L*) with time (*T*) using time after hatching as the independent variable and larval body length as the dependent variable. The coefficient of determination (*R*^2^), *F* value and *P* value all suggest that the equations determined by regression analysis have a high fit for the data.

**Table 3.** Equations, *F* values, *P* values and coefficient of determination (*R*^2^) of the relationship between the body length (*L*, mm) of *Chrysomya megacephala* larvae and the time after hatch (*T*, h) at seven constant temperatures.

Temperature (°C)	Equation	*F*	*P*	*R*^2^
16	*L* = −1.1E-6*T*^3^ + 5.1E-4*T*^2^ − 0.010*T* + 2.836	11411.1	<0.001	0.997
19	*L* = −3.0E-6*T*^3^ + 9.6E-4*T*^2^ − 6.7E-4*T* + 2.313	9824.1	<0.001	0.998
22	*L* = −5.0E-6*T*^3^ + 9.6E-4*T*^2^ + 0.062*T* + 2.319	4974.6	<0.001	0.996
25	*L* = −1.9E-5*T*^3^ + 0.003*T*^2^ + 0.046*T* + 2.048	5937.5	<0.001	0.998
28	*L* = −4.0E-5*T*^3^ + 0.005*T*^2^ + 0.059*T* + 1.961	1977.5	<0.001	0.995
31	*L* = −5.3E-5*T*^3^ + 0.005*T*^2^ + 0.091*T* + 2.014	1784.7	<0.001	0.996
34	*L* = −7.4E-5*T*^3^ + 0.006*T*^2^ + 0.120*T* + 2.093	3155.6	<0.001	0.998

The equations in Table 4 describe the time (*T*) changes with larval body length (*L*) using time after hatching as the dependent variable and larval body length as the independent variable. To obtain better curves and equations, only the larval change data from hatching to peak feeding stage is modelled by regression analysis. The coefficient of determination (*R*^2^), *F* value and *P* value all suggest that the equations obtained by regression analysis have a high fit after data elimination.

**Table 4.** Equations, *F* values, *P* values and coefficient of determination (*R*^2^) of the relationship between the time after hatch (*T*, h) and the body length of *Chrysomya megacephala* larvae (*L*, mm) at seven constant temperatures.

Temperature (°C)	Equation	*F*	*P*	*R*^2^
16	*T* = 0.125*L*^3^ − 3.832*L*^2^ + 52.398*L* − 99.977	4014.4	<0.001	0.994
19	*T* = 0.113*L*^3^ − 3.239*L*^2^ + 38.743*L* − 64.575	2169.8	<0.001	0.992
22	*T* = 0.046*L*^3^ − 1.192*L*^2^ + 17.993*L* − 32.748	1957.4	<0.001	0.994
25	*T* = 0.064*L*^3^ − 1.624*L*^2^ + 18.113*L* − 29.789	3534.0	<0.001	0.997
28	*T* = 0.066*L*^3^ − 1.714*L*^2^ + 17.728*L* − 31.257	646.5	<0.001	0.989
31	*T* = 0.042*L*^3^ − 1.116*L*^2^ + 12.502*L* − 21.198	383.7	<0.001	0.985
34	*T* = 0.049*L*^3^ − 1.352*L*^2^ + 14.820*L* − 39.324	309.3	<0.001	0.985

**Figure 4.**
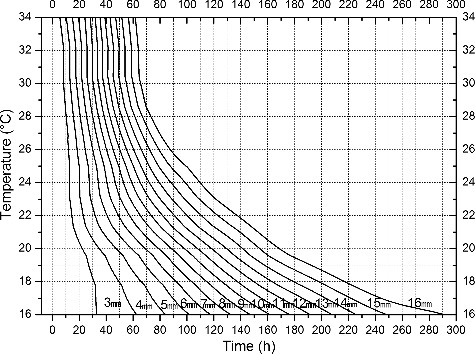
Isomegalen diagram of *Chrysomya megacephala* larvae from egg hatching to peak feeding stage. Time was plotted against temperature where each line represents developmental larval length in 3–16 mm, size indicated by number at the lower left of each contour.

The development data obtained was used to construct an isomegalen diagram (Figure 4). Using this development model, the corresponding larval age can easily be estimated based on larval body length at different temperatures up to peak feeding stage.

### Intra-puparial morphological changes over time

**Figure 5.**
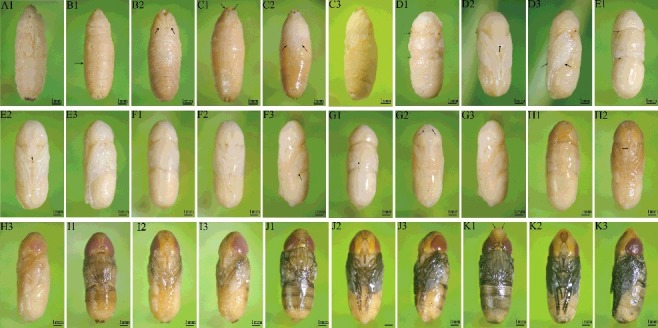
Intra-puparial morphological changes of *Chrysomya megacephala*. (A1) dorsal view of larvae – pupae dissociation stage; (B1) dorsal view of early cryptocephalic pupal stage, arrow indicates the larval-like body segmentation; (B2) ventral view of early cryptocephalic pupal stage, arrows indicate the emerged short legs and wing stubs; (C1) dorsal view of late cryptocephalic pupal stage, arrows indicate the emerged respiratory horns in the front end of pupa; (C2) ventral view of late cryptocephalic pupal stage, arrows indicate the elongated legs and wings; (C3) lateral view of cryptocephalic pupal stage; (D1) dorsal view of phanerocephalic pupal stage, arrows indicate the segmentation between head, thorax and abdomen remains unclear; (D2) ventral view of phanerocephalic pupal stage, arrow indicates the square-shaped mouthparts; (D3) lateral view of phanerocephalic pupal stage, arrows indicate the thick legs and wings; (E1) dorsal view of early yellow-eye stage, arrows indicate the segmentation into head, thorax and abdomen are clear; (E2) ventral view of early yellow-eye stage, arrow indicates the labellum is double-lobed; (E3) lateral view of early yellow-eye stage; (F1) dorsal view of middle yellow-eye stage; (F2) ventral view of middle yellow-eye stage, arrow indicates the elongated and narrowed mouthparts; (F3) lateral view of middle yellow-eye stage, arrow indicates the unfolded wings; (G1) dorsal view of late yellow-eye stage, arrow indicates the appeared thoracic dorsal bristles; (G2) ventral view of late yellow-eye stage, arrows indicate the development of antennae is completed but not coloured; (G3) lateral view of late yellow-eye stage; (H1) dorsal view of pink-eye stage; (H2) ventral view of pink-eye stage; (H3) lateral view of pink-eye stage; (I1) dorsal view of early red-eye stage; (I2) ventral view of early red-eye stage; (I3) lateral view of early red-eye stage; (J1) dorsal view of late red-eye stage; (J2) ventral view of late red-eye stage; (J3) lateral view of late red-eye stage; (K1) dorsal view of brown-eye stage, arrows indicate the emerged ptilinum; (K2) ventral view of brown-eye stage; (K3) lateral view of brown-eye stage.
B (early cryptocephalic pupal stage): the colour of puparium is brown red, and the pupa and puparium can be separated, but still fragile. It still appears shortened larva-like with rough surface and yellow-white colour (Figure 5(B1)). Short legs and wing stubs emerges, and the lengths of the legs are less than one-third of the body. Light colour respiratory horns emerge in the front end of pupa (Figure 5(B2,B3)).
C (late cryptocephalic pupal stage): pupa is yellow-white colour with smooth surface. Larval-like body segmentation can still be observed (Figure 5(C1)). Legs and wings elongate, length reaches half of the body. Respiratory horns colour darkened, still locates in the front of the pupa (Figure 5(C2,C3)).
D (phanerocephalic pupal stage): pupa is yellow-white colour. A transparent membrane on the surface forms, and is easy to peel from the puparium (Figure 5(D1–D3)). Head, thorax and abdomen begin to differentiate, but the segmentation between them remains unclear. Abdomen still has larval-like body segmentation (Figure 5(D1,D2)). Legs and wings are thick and further elongate, and length of the legs exceeds more than half of the body. The mouthparts are first observed as square-shaped (Figure 5(D3)).
E (early yellow-eye stage): pupa is yellow-white colour. The segmentation into head, thorax and abdomen is clear. The abdomen is smooth (Figure 5(E1)). The antennae become visible but are not fully developed, legs and wings are thinner. The labellum is double-lobed (Figure 5(E2,E3)).
F (middle yellow-eye stage): pupa is yellow-white colour (Figure 5(F1)). Antennae have obvious outline. Mouthparts are elongated and narrowed (Figure 5(F2)). The legs are thinner, and wings are unfolded (Figure 5(F3)).
G (late yellow-eye stage): pupa is yellow-white colour. Yellow-white thoracic dorsal bristles begin to appear. The abdomen is adult-liked segmented (Figure 5(G1)). The development of antennae and mouthparts is completed but not coloured (Figure 5(G2)). The legs are thin and wings are folded (Figure 5(G3)).
H (pink-eye stage): pupa is yellow colour. The compound eyes become pink (Figure 5(H1)). Cruciate bristles, thoracic dorsal bristles and maxillary palpi are light brown. Legs and antenna edges are light brown (Figure 5(H2,H3)).
I (early red-eye stage): pupa is yellowish-brown colour. The compound eyes are red. The bristles and hair in thorax and abdomen are dark brown (Figure 5(I1–I3)). The legs edges appear darkened with brown (Figure 5(I3)).
J (late red-eye stage): the compound eyes are red (Figure 5(J1)). Antennae are brown. Legs are black and the wings are light grey. The maxillary palpi are reddish brown. Thoracic dorsal bristles and the hair on abdomen are black (Figure 5(J1–J3)).
K (brown-eye stage): pupa is greyish-black colour. The antennae are brown. The legs are black and the wings are grey (Figure 5(K1–K3)). The colour of compound eyes further darkens to brown. Maxillary palpi are dark brown (Figure 5(K1)). The ptilinum begin to bulge (Figure 5(K1–K3)).

The pupal stage of *C. megacephala* is about 50% of the total immature stage. Aside from the first few hours after pupation, no colour or external morphological changes of the puparium can be seen to estimate the age; therefore, we observed the intra-puparial development of *C. megacephala* and categorized morphological changes into the following 11 sub-stages (A–K). Typical characteristics of each sub-stage are as follows:

A (pre-pupal stage): the formation of light coloured puparium, whose inner tissue and puparium are difficult to separate, and very easy to break during dissection. Pupa resembles a shortened larva with rough surface and yellow-white colour (Figure 5(A1)).

The time ranges of each sub-stage at different temperatures of *C. megacephala* are shown in Table 5.

**Table 5.** Intra-puparial development of *Chrysomya megacephala* related to time (h) at different temperatures.

	16 °C		22 °C		28 °C		34 °C	
Sub-stage	Min	Max	Min	Max	Min	Max	Min	Max
A	0	16	0	8	0	8	0	0
B	8	24	8	16	8	16	8	8
C	24	40	16	24	8	16	8	16
D	32	64	24	48	16	24	16	16
E	56	72	40	56	24	32	16	24
F	72	112	48	72	32	40	24	40
G	104	200	64	112	40	64	32	48
H	176	224	104	128	72	80	48	56
I	208	232	128	144	80	88	64	72
J	224	280	136	160	80	104	64	80
K	272	344	160	184	96	112	80	96

## Discussion

The development of *C. megacephala* has previously been reviewed by Richards and Villet [3] and Gruner et al. [21]. While in China, there are five development studies using the *C. megacephala* populations from different regions if our study is included (Table 6). Overall, *C. megacephala* from different regions have similar total development duration at same constant temperatures. However, total developmental durations of the colony in Chongqing [5] at lower temperature (16 °C and 19 °C) are much shorter than in Suzhou, where the Chongquing colony develops 6–8 days faster at each constant temperature. Interestingly, when comparing the results at higher temperatures (22 °C–34 °C), the results of present study and that of Yang et al. [5] are very similar. The difference at low temperatures is likely due to the Chongqing colony's adaptation to low temperature, as the Chongqing colony has a lower developmental threshold temperature *D*_0_. In addition, we find differences in the development duration between the colonies from China and America (Table 6). These discrepancies indicate the plasticity in developmental rates between different populations. However, studies have used different food and feeding methods, and this may also have an impact on the developmental duration. Future research is needed to rear and observe insects collected from different regions in the same laboratory using a standard method, combined with genomic analysis to determine whether the differences in developmental plasticity exist, and what genetic changes may underlie these differences.

**Table 6.** Developmental durations of *Chrysomya megacephala* from different regions.

Site and mean annual temperature	Total developmental duration	Site and mean annual temperature	Total developmental duration
Present study Suzhou, China (31° 2′ N, 120° 5′ E) 17 °C	16 °C (794.8 h, 33.1 d) 19 °C (533.2 h, 22.2 d) 22 °C (377.8 h, 15.7 d) 25 °C (280.8 h, 11.7 d) 28 °C (218.9 h, 9.1 d) 31 °C (190.8 h, 7.9 d) 34 °C (171.8 h, 7.2 d)	Yang et al. [5] Chongqing, China (28° 9′ N, 106° 9′ E) 18 °C	16 °C (594.0 h, 24.8 d) 19 °C (371.3 h, 15.5 d) 22 °C (310.2 h, 12.9 d) 25 °C (254.5 h, 10.6 d) 28 °C (209.6 h, 8.7 d) 31 °C (187.7 h, 7.8 d) 34 °C (181.5 h, 7.6 d)
Wang [32] Hangzhou, China (30° 3′ N, 120° 2′ E) 18 °C	16 °C (788.9 h, 32.9 d) 20 °C (405.1 h, 16.9 d) 24 °C (293.0 h, 12.2 d) 28 °C (228.2 h, 9.5 d) 32 °C (184.1 h, 7.7 d)	Ma et al. [29] Hangzhou, China (30° 3′ N, 120° 2′ E) 18 °C	18 °C (635.0 h, 26.5 d) 21 °C (390.9 h, 16.3 d) 24 °C (282.0 h, 11.8 d) 27 °C (237.1 h, 9.9 d) 30 °C (200.9 h, 8.4 d) 33 °C (190.1 h, 7.9 d)
Wang et al. [31] Guangzhou, China (23° 1′ N, 113° 2′ E) 22 °C	24 °C (272.4 h, 11.4 d) 28 °C (210.2 h, 8.8 d) 32 °C (200.4 h, 8.4 d)	Gruner et al. [21] Jacksonville, American (30° 2′ N, 81° 4′ W) Unknown	16.0 °C (719.2 h, 30.0 d) 21.2 °C (347.2 h, 14.5 d) 25.8 °C (221.1 h, 9.2 d) 30.8 °C (176.8 h, 7.4 d) 35.6 °C (164.7 h, 6.9 d)

The developmental threshold temperature *D*_0_ of *C. megacephala* calculated using the revised regression model proposed by Ikemoto and Takai [17] in this study is 11.41 °C. The result is similar with Richards and Villet [3], Yang et al. [5] and Gruner et al. [21], where all three studies show that *D*_0_ is about 10 °C. Interestingly, a development study using the *C. megacephala* colony from Guangzhou provides a different result [31]. The insects fail to complete development at 16 °C, indicating that the population from the warm and humid Guangzhou area has reduced capacity to complete development at lower temperatures and that the long-term geographical isolation may affect the low-temperature tolerance.

The maximum constant temperature we exposed insects to in this study is 34 °C, and *C. megacephala* can complete their developmental cycle under this temperature. Similarly, Gruner et al. [21] found that *C. megacephala* can develop at 35 °C, while the insects fail to complete their development at 40 °C. The results of Richards and Villet [3] show that *C. megacephala* could not complete development at 42.5 °C, and the survival rate of pupae at 37.5 °C is only 6.98%, indicating that 37.5 °C may be close to the highest temperature that *C. megacephala* can tolerate. Taken together, maximum resistance temperature of *C. megacephala* may be between 37.5 °C and 40 °C.
